# Secular trends in physical growth, biological maturation, and intelligence in children and adolescents born between 1978 and 1993

**DOI:** 10.3389/fpubh.2024.1216164

**Published:** 2024-04-29

**Authors:** Dominique A. Eichelberger, Aziz Chaouch, Valentin Rousson, Tanja H. Kakebeeke, Jon Caflisch, Flavia M. Wehrle, Oskar G. Jenni

**Affiliations:** ^1^Child Development Center, University Children's Hospital Zurich, Zurich, Switzerland; ^2^Department of Epidemiology and Health Systems, Quantitative Research, Center for Primary Care and Public Health (Unisanté), University of Lausanne, Lausanne, Switzerland; ^3^Children's Research Center, University Children's Hospital Zurich, Zurich, Switzerland; ^4^Department of Neonatology and Intensive Care, University Children’s Hospital Zurich, Zurich, Switzerland; ^5^Faculty of Medicine, University of Zurich, Zurich, Switzerland

**Keywords:** Flynn effect, cognitive functioning, IQ, height, weight, head circumference, skeletal maturation, birth weight

## Abstract

**Introduction:**

Human physical growth, biological maturation, and intelligence have been documented as increasing for over 100 years. Comparing the timing of secular trends in these characteristics could provide insight into what underlies them. However, they have not been examined in parallel in the same cohort during different developmental phases. Thus, the aim of this study was to examine secular trends in body height, weight, and head circumference, biological maturation, and intelligence by assessing these traits concurrently at four points during development: the ages of 4, 9, 14, and 18 years.

**Methods:**

Data derived from growth measures, bone age as an indicator of biological maturation, and full-scale intelligence tests were drawn from 236 participants of the Zurich Longitudinal Studies born between 1978 and 1993. In addition, birth weight was analyzed as an indicator of prenatal conditions.

**Results:**

Secular trends for height and weight at 4 years were positive (0.35 SD increase per decade for height and an insignificant 0.27 SD increase per decade for weight) and remained similar at 9 and 14 years (height: 0.46 SD and 0.38 SD increase per decade; weight: 0.51 SD and 0.51 SD increase per decade, respectively) as well as for weight at age 18 years (0.36 SD increase per decade). In contrast, the secular trend in height was no longer evident at age 18 years (0.09 SD increase per decade). Secular trends for biological maturation at 14 years were similar to those of height and weight (0.54 SD increase per decade). At 18 years, the trend was non-significant (0.38 SD increase per decade). For intelligence, a positive secular trend was found at 4 years (0.54 SD increase per decade). In contrast, negative secular trends were observed at 9 years (0.54 SD decrease per decade) and 14 years (0.60 SD decrease per decade). No secular trend was observed at any of the four ages for head circumference (0.01, 0.24, 0.17, and − 0.04 SD increase per decade, respectively) and birth weight (0.01 SD decrease per decade).

**Discussion:**

The different patterns of changes in physical growth, biological maturation, and intelligence between 1978 and 1993 indicate that distinct mechanisms underlie these secular trends.

## 1 Introduction

For over 100 years, substantial upward trends have been observed in a range of human traits, skills, and performances. Typical examples of these secular trends include the substantial gain in life expectancy since the beginning of the 19th century (1), the large generational increases in human growth characteristics such as body height and weight as well as the acceleration in biological maturation (2), the improved physiological strength as measured in sport achievements over many decades (1), and the rise in populations’ average intelligence since 1900 (3, 4). This secular trend was named the Flynn effect after James Flynn (5).

Environmental factors have frequently been proposed as the main cause of the positive secular trends in human characteristics; this includes the improved quality of nutrition, sanitation, hygiene and health care, the decreased rate of infections and other diseases, the higher value and better structure of educational systems, the reduced size of families and higher standard of child rearing, the increased overall complexity of environments, and ultimately, the economic advance of societies (6). In contrast to these environmental explanatory factors, increases in genetic diversity and consequently genetic advantages resulting from demographic trends towards more random mating in recent societies were also proposed (7). However, a single explanation does, likely, not suffice to fully account for the positive secular trends in populations’ traits but a combination of multiple aspects that may vary across domains may be relevant (3).

In the most recent decades, secular trends in a number of traits have been observed to slow, stop, and even reverse. For example, in the Northern European countries, the rise in adult height has reached a plateau (8), and intelligence test scores have even declined over time (9). The slowing and flattening of secular trends since the end of the 20th century may be explained by the fact that in industrialized populations with the highest living standards, an increasing proportion of individuals have reached the upper limit of their genetic and biological potential, and consequently, the benefits of environmental factors are now fading (1, 9). Notably, a reverse in the direction of secular trends (i.e., negative secular trends) is expected to stem from a different set of processes because the factors that previously contributed to an increase are unlikely to explain a subsequent decline (10).

Mingroni was among the first who did not only focus on single secular trends, but also explored secular changes in multiple variables such as height, head circumference, intelligence, myopia, asthma, autism and others at the same time (7, 11). In fact, the concurrent investigation of secular changes in multiple traits may contribute to identifying the explanatory processes underlying them. More specifically, if a similar timing is apparent in multiple traits, likely, a common mechanism underlies the respective secular trends. Conversely, if the timing is different for different traits, this may suggest the effect of different factors. Particularly, the comparison of secular trends in growth with maturational and intelligence parameters is interesting because they have all been attributed to improved nutrition and health care [see, e.g., (12)], while other authors have postulated that improved education and increased overall complexity of environments have led to the changes of the intellectual capacity across generations [see, e.g., (13)]. However, to date very few studies have compared secular trends in multiple traits concurrently in the same cohort. Notable exceptions are the studies of conscripts in Northern European countries. In fact, Sundet et al. (14) showed that the increase in intelligence and height followed an identical pattern among Norwegian military draftees between 1950 and 1990. However, the increase in height was driven primarily by the upper half of the height distribution, while the lower part of the IQ distribution was responsible for the IQ gain. Rönnlund et al. (15) also found parallel secular trends in the two domains between 1970 and 1979, but the rise in intelligence test scores continued during 1980–1993, when the secular trend in height was no longer observed. Therefore, both studies concluded that improved nutrition, better medical care, and perhaps other biological factors might not have been the primary cause for the Flynn effect, because secular trends in growth were not observed in the same periods and to the same extent.

Previous findings on secular trends in traits assessed concurrently in the same cohort are limited to adults, mainly conscripts aged 18–20 years old (14–18). However, secular trends have been suggested to vary with age. In the Netherlands, for example, Woodley and Meisenberg (19) found that intelligence test scores between 1950 and 1990 increased more among 30-year-olds than 5- to 6-year-olds, and they even decreased among 14- to 16-year-olds, indicating a negative trend. However, findings about age-specific secular trends in intelligence remain inconsistent, in particular in recently published meta-analyses: Whereas Pietschnig and Voracek (3) reported larger secular trends in intelligence in adults than in children and adolescents, Trahan et al. (4) could not confirm these findings. One reason for the discrepancies between studies may be the frequent inclusion of cross-sectional studies, which are prone to sample composition effects.

Larger secular trends for height have been reported during childhood than during adulthood, with a maximum at puberty (2, 20, 21). These age-related differences suggest that beyond environmental factors, maturational tempo may contribute to secular trends in growth parameters during development (2, 21–26). In fact, several studies have confirmed a secular trend in skeletal maturity, a biological indicator of the tempo of growth and maturation (27, 28).

The present study aims to advance the understanding of similarities and dissimilarities in the timing of secular trends in growth parameters including height, weight, and head circumference, as well as in biological maturation (measured by bone age), and intelligence by assessing these traits longitudinally during four developmental phases, at ages of 4, 9, 14, and 18 years, in a single cohort born between 1978 and 1993.

## 2 Methods

### 2.1 Sample

The data originates from the Zurich Longitudinal Studies (ZLS), a set of three cohort studies that examined physical, motor, and mental development and social environment from birth to young adulthood (29). The third ZLS cohort (ZLS-3) covers the largest range of birth years, from 1973 to 2002, and consequently was considered for the current analyses. Individuals included in the ZLS-3 cohort are the offspring of participants of the first ZLS cohort (ZLS-1). ZLS-1 participants were enrolled into the ZLS between 1954 and 1961 as a representative sample of the population of Zurich with regard to parental occupation (30) and if they were born into a Swiss family residing in Zurich. Consequently, all participants in the ZLS-3 cohort have at least one Swiss parent. In total, 295 infants from 161 families were enrolled at birth.^1^ The current analyses only included those who did not have a developmental disability or genetic syndrome with known effects on intellectual ability or growth and who were fluent in German, because intelligence testing was conducted in German.

The vast majority of the ZLS-3 cohort, 92.2%, was born between 1978 and 1993, with less than five children being born each year outside of this range. We therefore restricted the analyses to the 236 children and adolescents from 132 families born during this period (Supplementary Figure S1). We report the results of a sensitivity analysis that incorporated participants born before 1978 and after 1993 in Supplementary material S1.

### 2.2 Assessments

Data on child development were assessed numerous times between birth and 18 years of age. All developmental assessments were conducted by a psychologist or a developmental pediatrician at the University Children’s Hospital Zurich, Switzerland. For the current analyses, examinations at the ages of 4, 9, 14, and 18 years were used because they included the assessment of height, weight, head circumference, biological maturation, as well as intellectual abilities [see (29) for details on the full study protocol].

Height was measured in the standing position to the nearest millimeter with a stadiometer. For the weight measurement, the children stood on a beam scale with an accuracy of 0.1 kilograms. Head size was measured at the widest possible circumference of the head: encompassing the broadest part of the forehead above the eyebrow, above the ears, and the most prominent part of the back of the head. The head measurement was taken three times, and the largest measurement to the nearest millimeter was selected. A more detailed description of the measurement of height, weight, and head circumference can be found in Prader et al. (31).

Biological maturation was quantified as skeletal maturity (32) and measured by bone age at ages 14 and 18 years. Bone age was analyzed from hand x-rays of the left hand using the software BoneXpert (33, 34).

Intellectual abilities were assessed using age-appropriate and standardized full-scale IQ instruments: at 4 years the Snijders–Oomen Non-Verbal Intelligence Test [SON; (35)] and at 9, and 14 years, the Wechsler-Intelligence Scale for Children-Revised [WISC-R; German version; (36, 37)] was used. The SON is a non-verbal intelligence test based on five sub-tests (sorting, mosaic, combination, memory, and copying) and is normed for children aged 2.5 to 7 years. The WISC-R comprises six subtests to estimate verbal IQ (information, comprehension, arithmetic, similarities, vocabulary, and digit span) and five subtests to estimate performance IQ (coding, picture completion, picture arrangement, block design, and object assembly). The full-scale IQ is estimated from the verbal and the performance IQ. The WISC-R is normed for children aged 6 to 16 years.

Additionally, birth weight was collected from the birth report and used as an indicator of prenatal conditions. Childhood socioeconomic status (SES) was estimated from parental occupation and maternal education at ages 1 or 3 months on a scale ranging from 2 to 10 with higher scores indicating lower SES, following Largo et al. (38).

### 2.3 Statistical analysis

We defined the secular trend as the effect of the year of birth on height, logarithm of weight, head circumference, biological maturation, and full-scale IQ among participants of the same age after conditioning on sex and SES. The year of birth was considered as a continuous variable with decimals so that, for instance, a child born on 26.04.1985 had a year of birth equal to 1985.315. Because the IQ tests were not the same at each age, we analyzed each age group separately. For the sake of comparability, we also analyzed data from the other outcomes separately in each age group.

We use $y_{\mathrm{ij}}$ to denote the outcome observed on child i at age j= 1 (4 years), j= 2 (9 years) or j= 3 (14 years); $t_{i}$ the year of birth of child i; ${\mathrm{sex}}_{i}$ the sex (female = 0, male = 1) of the child; and ${\mathrm{ses}}_{i}$ their SES. We consider this regression model,


$$\begin{array}{l} y_{\mathrm{ij}}=\beta_{0j}+\beta_{1j}\left(\frac{t_{i}-1985}{10}\right)+\beta_{2j}{\mathrm{sex}}_{i}+\beta_{3j}\left({\mathrm{ses}}_{i}-5\right)\\ \;\;\;\;\;\;\;\;\;\;\;\;\;\;\;\;\;\;\;\;\;+\beta_{4j}\left({\mathrm{ses}}_{i}-5\right)\left({\mathrm{ses}}_{i}>5\right)+\varepsilon_{\mathrm{ij}} \end{array}$$

where $\beta_{j}={\left(\beta_{0j},\beta_{1j},\beta_{2j},\beta_{3j},\beta_{4j}\right)}^{T}$ is a vector of regression coefficients and$\varepsilon_{\mathrm{ij}}$ is a residual error term with mean zero and variance $\sigma_{j}^{2}.$ Note that in this model, $\beta_{0j}$ is a global intercept term and $\beta_{1j}$ quantifies the contrast between two cohorts of age j with same sex and SES born 10 years apart, expressed in original outcome units. Additionally, $\beta_{2j}$ and $\beta_{3j}$ refer to the sex and linear SES effect on the outcome, respectively, and $\beta_{4j}$ allows SES to have a different linear effect on the outcome below and above the median value of 5. This last coefficient was included in the model only when statistically significant (*p* < 0.050). A model including an interaction term between the year of birth and sex, thus allowing for different secular trends in males and females, was also tested, but the interaction term was never found to be statistically significant. Therefore, we report results obtained for the simpler model without such interaction. Because study participants were nested within 132 families, error terms from individuals belonging to the same family were allowed to be correlated using an exchangeable covariance structure. We used Generalized Estimating Equations (39) fitted separately at each age to obtain estimates of the vector of regression coefficients $\beta_{j}$ and the residual variance $\sigma_{j}^{2}.$ The same procedure was applied for birth weight.

Comparing secular trend estimates for outcomes measured on different scales requires such estimates to be reported on a relative (i.e., unitless) scale. Two types of standardization can be used for this purpose: an external out-of-sample standardization or an internal in-sample one. In this study, we used an internal standardization with the relative secular trend at age j defined as the ratio $\beta_{1j}/\sigma_{j}$. This ratio quantifies the average change in the outcome between two cohorts born 10 years apart as a function of the in-sample within-cohort inter-individual variability after controlling for sex and SES differences. Inference was performed by approximating the variance of the ratio $\beta_{1j}/\sigma_{j}$ using the Delta method (40). The relative secular trend estimate can be interpreted as a regular standardized difference in means [Cohen’s d; (41)], with values of 0.2, 0.5, and 0.8 referring to small, moderate, and large effects, respectively. We direct interested readers to the Supplementary material S2 for a comparison of results obtained with internal and external standardizations.

All statistical analyses were carried out in R version 4.2.1 (42) using a level of statistical significance of 5%.

### 2.4 Ethics

The study was reviewed and approved by the Ethical Committee of the Canton of Zurich, Switzerland (Basec-Nr. 2018-00686); further details on the informed consent procedure since the initiation of the ZLS in 1954 are provided in Wehrle et al. (29).

## 3 Results

Table 1 provides a descriptive summary at the four assessment time-points of the participant characteristics: sex distribution, SES, growth parameters, bone age, and IQ score. Between the ages of 4 and 18 years, 35 participants (14.8%) dropped out of the study.

**Table 1:** Demographic information, IQ scores, and growth parameter at the four assessment time-points, for participants born between 1978 and 1993.

	4 Year	9 Year	14 Year	18 Year
Number of participants (*N*)	229	218	205	200
Males [*n* (%)]	120 (52%)	114 (52%)	108 (53%)	102 (51%)
Family SES (Mdn [IQR])	5 [4, 6]	5 [4, 6]	5 [4, 6]	5 [4, 6]
Birth weight [g]; M (SD)	3289 (434)	3282 (431)	3273 (426)	3264 (412)
Head circumference [cm]; M (SD)	50.7 (1.3)	52.8 (1.3)	54.9 (1.5)	56.2 (1.7)
Height males [cm]; M (SD)	104.2 (4.0)	134.2 (5.4)	163.6 (8.4)	177.4 (6.7)
Height females [cm]; M (SD)	102.9 (3.5)	133.7 (4.7)	161.8 (5.3)	165.5 (4.8)
Weight males [kg]; M (SD)	16.8 (1.9)	28.9 (4.6)	52.2 (10.9)	69.7 (11.7)
Weight females [kg]; M (SD)	16.1 (1.7)	29.2 (5.2)	51.5 (8.9)	58.7 (8.4)
Bone age [years]; M (SD)	Not available	Not available	13.9 (1.1)	17.5 (0.9)
Full scale IQ; M (SD)	116.1 (11.4)	104.1 (11.3)	111.3 (10.8)	Not available

Mdn, median; IQR, interquartile range; M, mean; SD, Standard Deviation; SES, socioeconomic status based on a composite score of parents’ education and occupation, with scores ranging from 2 to 10 (higher scores indicating a lower SES).

**Figure 1:**
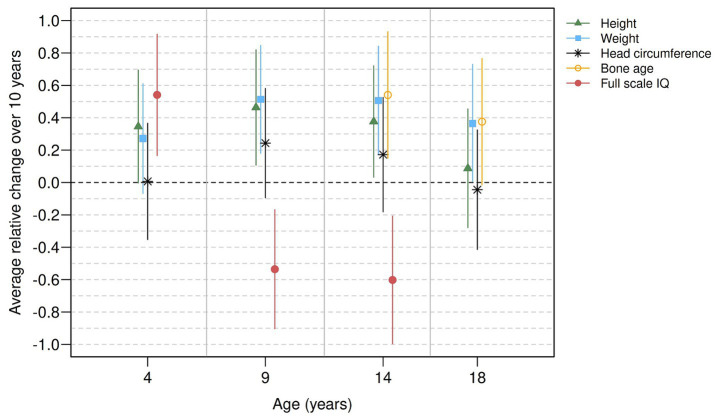
Relative secular trend estimates for height, weight, head circumference, bone age and full scale IQ at four different ages (4, 9, 14, and 18 years). The average relative change over 10 years can be interpreted as a standardized difference in means [Cohen’s d; (41)], with values of 0.2, 0.5, and 0.8 referring to small, moderate, and large effects, respectively.

Table 2 and Figure 1 present the relative secular trend estimates of height, weight, head circumference, biological maturation, and intellectual abilities at ages 4, 9, 14, and 18 years with 95% confidence intervals (CI).

**Table 2:** Relative secular trend estimates (with 95% confidence interval) for height, weight, head circumference, bone age, and full scale IQ at four different ages (4, 9, 14, and 18 years).

	Age 4 years	Age 9 years	Age 14 years	Age 18 years
Height	0.35 (0.00; 0.69)	0.46 (0.11; 0.82)	0.38 (0.03; 0.72)	0.09 (−0.28; 0.45)
Weight	0.27 (−0.07; 0.61)	0.51 (0.18; 0.85)	0.51 (0.17; 0.84)	0.36 (0.00; 0.73)
Head circumference	0.01 (−0.35; 0.37)	0.24 (−0.09; 0.58)	0.17 (−0.18; 0.53)	−0.04 (−0.41; 0.32)
Bone age	Not available	Not available	0.54 (0.15; 0.93)	0.38 (−0.01; 0.77)
Full scale IQ	0.54 (0.17; 0.92)	−0.54 (−0.90; −0.17)	−0.60 (−1.00; −0.21)	Not available

The estimates refer to the average change in the outcome value between two cohorts born 10 years apart divided by the within-cohort standard deviations, after controlling for sex and SES.

The secular trends in height and weight at age 4 years were positive (0.35 SD increase per decade for height and a statistically insignificant 0.27 SD increase per decade for weight, respectively) and remained about the same at ages 9 and 14 years (for height: 0.46 SD and 0.38 SD increase per decade; for weight: 0.51 SD and 0.51 SD increase per decade, respectively). Weight at age 18 years also remained similar (0.36 SD increase per decade). In contrast, the secular trend in height was no longer evident at age 18 (0.09 SD increase per decade). For head circumference, no statistically significant secular trends were found for the four developmental periods (0.01, 0.24, 0.17, and − 0.04 SD increase per decade for 4, 9, 14, and 18 years, respectively). For birth weight, no secular trend was found (0.01 SD decrease per decade). Secular trends for biological maturation at 14 years were similar to those of height, and weight (0.54 SD increase per decade), and remained positive at age 18 years (statistically insignificant 0.38 SD increase per decade).

Statistically significant secular trends for intellectual abilities were found for all three available age periods: At 4 years, the secular trend was positive, with a 0.54 SD. However, negative trends were observed at 9 years (0.54 SD decrease per decade) and at 14 years (0.60 SD decrease per decade). The estimates for verbal and performance IQ assessed with the WISC-R at 9 and at 14 years indicated a negative secular trend for performance IQ at 9 years (0.61 SD decrease per decade) and for verbal IQ at 14 years (0.85 SD decrease per decade) whereas the negative secular trend estimates for verbal IQ at 9 years and performance IQ at 14 years were statistically insignificant (see Supplementary Table S1 for details).

Secular trend estimations for height and weight at 14 years became non-significant when adjusted for biological maturation (quantified as bone age at 14 years) while the positive secular trend remained similar for IQ (Supplementary Table S2).

## 4 Discussion

The aim of these analyses was to examine secular trends of growth measures, biological maturation, and intelligence concurrently during four developmental phases: at 4, 9, 14, and 18 years. The analyses examined data from participants of the third cohort of the ZLS, who were born between 1978 and 1993 and assessed between 1982 and 2011 [see (29) for details on the ZLS cohorts]. Notably, this is the first study to investigate secular trends in several traits during four phases of child development in a single cohort. Positive secular trends were observed in height and weight at 4, 9, and 14 years. In contrast, at age 18 years, the secular trend of weight remained similar while the secular trend of height became negligible. No secular trends were observed in head circumference at any of the ages or in birth weight. A secular trend in bone age was apparent at the age of 14 years while it was only marginally significant at 18 years. For intelligence, a positive secular trend was apparent at age 4 years. In contrast, a negative secular trend was found at ages 9 and 14 years.

A positive secular trend in height was found in this cohort of Swiss children and adolescents. In contrast, no secular height trend was observed at 18 years, which is in agreement with Vinci et al. (43). These authors described a steady increase in the height of young Swiss adults after 1937 which slowed and nearly stopped at an average height of 178 cm for men and 166 cm for women after 1970. A possible reason for the ongoing secular trend in children and adolescents is that such changes are still present in the pubertal growth spurt in adolescence and in the developmental tempo during childhood (21, 44). In fact, we also found a secular trend in skeletal maturity as measured by bone age, which is considered a reliable indicator of the maturational tempo of growth (32). Positive secular trends in bone age measures between the 1960s and 2000 have been reported in two previous studies from Taiwan and South Africa (27, 28). The pattern in the ZLS-3 of a positive secular height trend in childhood and adolescence in the absence of such a trend in young adulthood may be due to the fact that children and adolescents born in more recent years (1990s) were biologically more mature (i.e., showed earlier maturation) compared to individuals born longer ago (1970s).

The positive secular trends that we found for weight in the current study are in line with those found in the adult population in Switzerland (45). This likely reflects the growing obesity epidemic worldwide (46), including Switzerland (45). Only since about 2012, after the cohort studied here reached adulthood, has the secular trend in children’s weight leveled off (8, 47, 48).

In line with recent studies, we found no evidence for a secular trend in birth weight, an important parameter of child growth *in utero*. Birth weight was monitored in Norway in registry studies between 1860 and 1984. It declined from 1860 to 1900 and increased again by about 150 g between 1900 and 1940, but it has remained largely constant since then (49). Birth weight has also changed little over recent decades in other countries (49–51). In contrast to a number of previous studies that have shown an increase in head circumference increased in recent decades [see (52) for a review], we did not observe a secular trend in head circumference.

Together, the findings of positive secular trends at most developmental phases in height, weight and biological maturation, of no secular trends in head circumference as well as in birthweight and the differential secular trends at different developmental phases in intelligence make it unlikely that any single environmental factor is the primary driver of these changes.

The hypotheses that have been put forward to explain the positive secular trends in various traits across the 20th century include, among others, improved nutritional quality and quantity [i.e., “nutrition hypothesis” advocated by Lynn (12, 53, 54)], better health and health care (55), and advances in education and the educational system (56–58). It has been argued that high-income countries, including Switzerland, are currently approaching an asymptote: with environmental improvements having unfolded over past decades, large parts of the population have now reached their genetic and biological potential, and any further improvements in those environmental conditions may no longer result in an upwards trend in those traits (1, 9). This “environmental saturation effect” was formalized by Irving Gottesman’s genome-environment interaction model (59), postulating a maximum developmental potential that can be realized to a large extent if living conditions are favorable. In fact, such an effect may be reasonably assumed to underlie the secular trends in some of the growth parameters assessed in the current study, most notably in height (i.e., no secular trend in adult height measured at 18 years) and head circumference (i.e., no secular trend at any developmental phase).

Exploring the reasons for the secular trend pattern in IQ that we found in the current study is challenging: Differential secular trends became apparent at 4 years (positive trend) and at 9 and 14 years (negative trends). To thoroughly discuss this finding, first, a methodological issue must be considered, namely, the use of different instruments. At 4 years, the SON was used, a nonverbal test, providing a single IQ score, without any further distinction into different IQ domains (13). In contrast, at 9 and 14 years, the WISC-R was used, and its full-scale IQ combines estimates of verbal and performance IQ (36, 37). Previously, larger IQ gains have been described for fluid or performance IQ than for crystallized or verbal IQ (3). This raises the question whether the different aspects of IQ captured by the SON and the WISC-R could explain the differences in the secular trend patterns. When separately investigating the secular trends in verbal and performance IQ at 9 and 14 years in the current study, a negative trend was observed in both IQ domains, although only reaching statistical significance for performance IQ at 9 years and for verbal IQ at 14 years. Performance IQ may be considered conceptually overlapping with fluid IQ, including some very similar subtests (e.g., mosaics and block design in the SON and the WISC-R, respectively). This suggests the differential directions of the secular trends at 4 years (positive trend assessed with SON) versus at 9 and at 14 years (negative trend assessed with WISC-R) are, likely, not explained solely by the different tests used at different developmental periods. Alternative lines of explanation are discussed in the following.

The *positive* secular trend in intelligence at 4 years may have resulted from changes in child rearing and parenting from the 1970s onwards. This idea was summarized by Rodgers and O’Keefe (60) in their integrative theory termed ‘Parental Executive Model’. Their theory posits that parents are in general highly motivated to facilitate their children’s intellectual development, and consequently that they use resources available to them, namely, those that have previously been proposed by other theories to explain the Flynn effect, such as improved nutrition, education, technology, healthcare, and so forth. Additionally, a cross-generational feedback loop exists, whereby children who are “better nurtured for intelligence” (10, p. 5) have improved abilities and gain access to progressively superior resources, thereby amplifying their own children’s intellectual growth. Several studies have, indeed, provided evidence for a parental contribution to secular trends in IQ (61–64). Swiss parents have likely also become increasingly involved in supporting their children’s development and have created more stimulating and nurturing environments for their young children, leading to the positive secular trend in early childhood that was found in the current study.

The *negative* secular trend that we found in 9- and 14-year-olds is consistent with recent studies, especially in the European Nordic countries [see, e.g., (9) for a general review]. Although two large meta-analyses have reported average Flynn effects of about three IQ points per decade since the 1930s (3, 4), the secular trend in intelligence has not always been linear, and seems to have slowed so much over the last 30 years that it has even become negative. A systematic review in 2016 described this reverse Flynn effect in seven European countries among individuals tested between 1975 and 2012 (9). A more recent review found secular IQ declines in even more countries in Africa, Europe, and North and South America (65).

The Netherlands are often described as the country that experienced an IQ decline as early as 1975 [e.g., (19)]. In fact, analysis of secular trends in this country between 1968 and 2005 showed a pattern very similar to the findings of our study; although IQ was quite stable among preschoolers, a negative secular trend emerged among the 14-year-olds (19). While improvements in education may have contributed to raises in IQ in the past (56–58), the IQ decline observed in our cohort (and in other’s) is unlikely to be attributable to changes in the educational structures in Switzerland as they have improved over the course of the 20th century rather than worsened. For example, the average class size declined steadily from 55 to 20 students per class between 1870 and 1980 (66, 67). Also, the sharp decline within a relatively short period of time cannot be attributed to a poorer educational system. Similarly, other factors related to the living standard that may have previously contributed to the positive secular trends in IQ, such as improved nutrition, hygiene status or the medical care system (1), are unlikely to have declined in Switzerland since the mid-1970s, when the participants of the current study were born. Thus, they cannot explain the negative secular trend in IQ seen in the current study at 9 and 14 years. In fact, Rodgers argued that the same factors contributing to positive secular trends may be implicated in the slowing and flattening of these trends, however, they certainly cannot be responsible for their turn-around. Rather, an entirely different set of explanations may require consideration (10).

One new explanation that has been brought forward to explain the observed negative secular trends in IQ in recent decades is that of dysgenic effects: disadvantageous genetic effects of higher reproduction rates of low-ability as opposed to high-ability individuals within populations (68–70). However, this cannot be responsible for the negative secular trends observed in 9- and 14-year-olds because of the positive trend at 4 years in the identical cohort. In any case, selection-driven genetic changes in the population of the ZLS-3 are implausible considering the relatively short time span and the large magnitude of the effect. Moreover, because the sample is relatively homogenous and born in the greater Zurich area, changes in the sample composition through immigration or methodological artifacts due to, for instance, recruitment biases between cohorts can be excluded.

It has further been suggested that fluid and crystallized IQ undergo differential secular trends (e.g., illustrated in 3, Figure 1) and that the respective divergence may contribute to varying trend patterns (10). While our data showed no differential effect for verbal and performance IQ, it cannot contribute to this exact discussion as the assessment instruments are not designed to distinguish between fluid and crystallized IQ. Further studies are needed to systematically investigate this, alongside identifying other potential explanatory processes underlying the differential patterns of secular trends across different traits, including IQ.

As already outlined above, the ultimate cause of secular trends suggested by Flynn (13)—the industrial revolution, economic progress, educational standards, and the complexity of society—may have reached a maximum since the 1980s, at least in developed countries, and the upper limit of children’s developmental and intellectual capacities may have been reached in recent decades. Consequently, any further changes in the environment may now result in a weakening (i.e., slowing/flattening) or in random increases and declines of secular trends.

## 5 Strengths and limitations

A particular strength of this study is the use of longitudinal data with a consistent sample composition over time. Furthermore, the use of in-sample standardization when defining secular trend estimates enhanced the comparability of estimates across traits because the within-cohort variability $\sigma_{j}$ at age j is not affected by secular trends and is estimated in the same sample for all traits.

The current study is limited by a relatively small sample size and a limited range of birth years between 1978 and 1993. However, previous studies were also able to identify secular trends in various traits across comparable ranges [e.g., (17, 71, 72)]. The cohort included in the current analyses (ZLS-3) consists of the offspring of the ZLS-1 cohort. This unique study setting makes it challenging to replicate the findings in an independent sample. Further, the assessment of IQ with different tests at different developmental phases complicates the comparison of the full-scale IQs across ages. Importantly, however, comparing the overlapping concepts captured by the different tests confirmed the differential pattern of secular trends at 4 versus 9 and 14 years of age. Due to the design of the IQ tests applied in the current cohort, the data cannot contribute to the body of research on differences in secular trends in fluid and crystallized intelligence that have been consistently reported previously (3).

## 6 Conclusion

The current study provides insights into the secular trends of physical growth measures, biological maturation, and intelligence at 4, 9, 14, and 18 years of age over a period of 15 years. The differing patterns in the secular trends of these psychological and biological traits suggest that their etiology may vary between traits and development phases. The findings may also have important implications for clinical studies. For example, when investigating the development of at-risk groups, such as children born preterm or those raised in socio-economically disadvantaged conditions, it is crucial to use same-aged control groups from similar birth years. Such an approach is critical to ensure that interpretations of cohort differences (e.g., due to improved medical care or after interventions) are not confounded by secular trends [e.g., (73, 74)]. Overall, the findings of the current analyses highlight the need for further research to monitor secular trends of human characteristics.

## Data availability statement

The raw data supporting the conclusions of this article will be made available by the authors, without undue reservation.

## Ethics statement

The studies involving humans were approved by Ethical Committee of the Canton of Zurich, Switzerland (Basec-Nr. 2018-00686). The studies were conducted in accordance with the local legislation and institutional requirements. Written informed consent for participation in this study was provided by the participants’ legal guardians/next of kin.

## Author contributions

DE contributed to the formulation and development of the research goals, conceived and designed the analysis of the data samples, contributed to data collection of some samples, drafted and edited the manuscript. AC performed the statistical analysis of the entire data set, drafted and edited the manuscript. VR supervised the statistical analysis of the data, reviewed and edited the manuscript. TK contributed to the formulation and development of the research goals, and reviewed and edited the manuscript. JC contributed to the formulation and development of the research goals, performed data collection of the children and adolescents, reviewed, and edited the manuscript. OJ is the principal investigator of the Zurich Longitudinal Studies (ZLS), contributed to the formulation and development of the research goals, and drafted and edited the manuscript. FW is the current project leader of the ZLS, contributed to the formulation and development of the research goals, and drafted and edited the manuscript. All authors contributed to the article and approved the submitted version.

## Footnotes

^1^This number differs from that published in Wehrle et al. (29) (*N* = 327) because subsequent data checks revealed that 31 participants had incorrectly been listed as enrolled at birth. No data had been assessed in these individuals. One participant declined the continued use of the childhood data for future research projects and is thus no longer considered part of ZLS-3.
