# Supplementary material for: Secular trends in physical growth, biological maturation, and intelligence in children and adolescents born between 1978 and 1993
Source: Front Public Health. 2024 Apr 29;12:1216164. doi: 10.3389/fpubh.2024.1216164 (PMC11089810; doi:10.3389/fpubh.2024.1216164)
Supplement: Supplementary file 1 [file Data_Sheet_1.pdf]

Supplementary Material

Secular trends in physical growth, biological maturation, and intelligence in children and adolescents born between 1978 and 1993

Dominique A. Eichelberger, Aziz Chaouch, Valentin Rousson, Tanja H. Kakebeeke, Jon Caflisch, Flavia M. Wehrle, and Oskar G. Jenni*

*** Correspondence:** Oskar G. Jenni, MD, Child Development Center, University Children's Hospital Zurich, Zurich, Switzerland, Email: oskar.jenni@kispi.uzh.ch

1 Supplementary Material S1: Sensitivity Analysis; Effect of Including Participants Born Before 1978 or After 1993 on the Relative Secular Trend Estimates.

The third cohort of the Zurich Longitudinal Studies (ZLS-3; 1) included participants born between 1973 and 2002. However fewer than five participants were born each year between 1973 and 1977 and between 1994 and 2002 (see Supplementary Figure S1). Because years of birth at the tails of the distribution can potentially have a strong impact on the secular trend estimation by acting as leverage points, the main analysis was conducted on the bulk of participants, those born between 1978 and1993. In this sensitivity analysis, we assess the impact of gradually including birth years before 1978 or after 1993 on the secular trend estimates, with those estimates obtained from the bulk of participants serving as the reference.

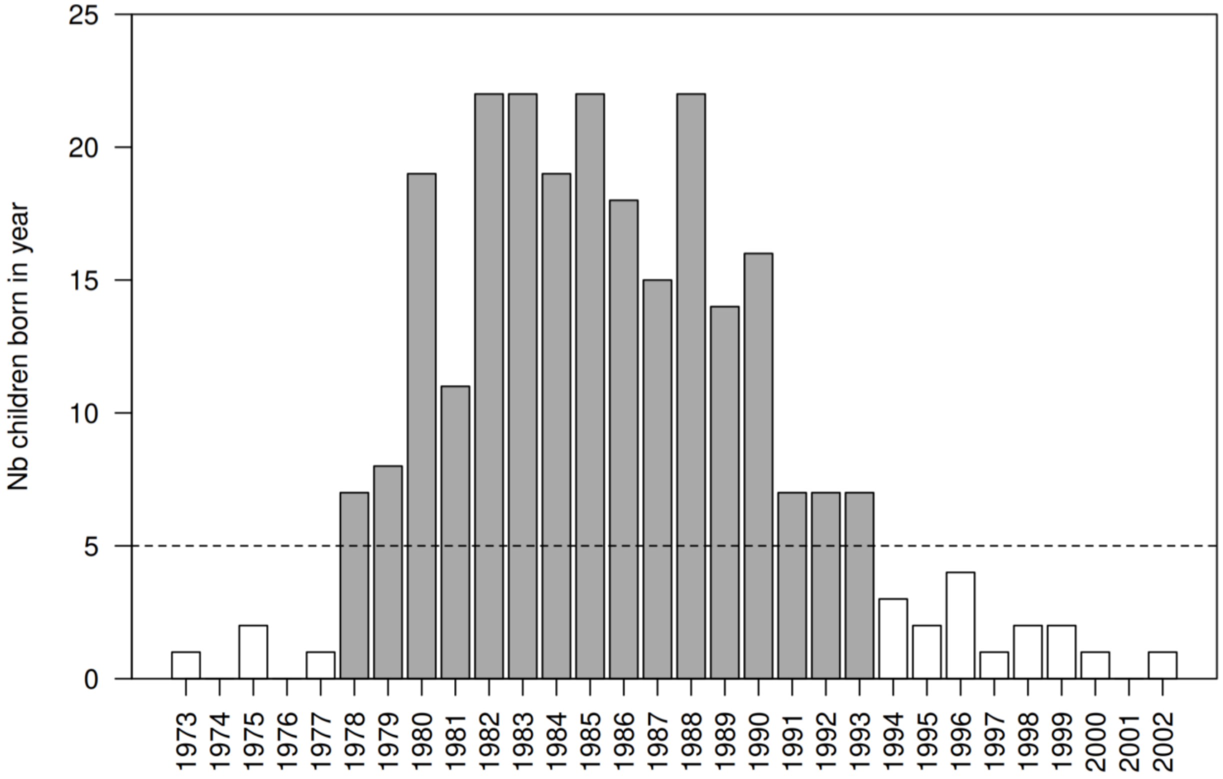

Supplementary Figure S1. Number of participants of the third cohort of the Zurich Longitudinal Studies (ZLS-3; 1) born each year between 1973 and 2002. The gray bars represent birth years that were included in the main analysis as they consist of more than five individuals per year (as indicated by the dashed line). The white bars indicate birth years that were excluded from the main analysis due to low birth counts along with an increased risk of being leverage points (see Supplementary Figure S2).

Given the model described in the methods section, let $\theta_j = \beta_{1j}/\sigma_j$ refer to the internally standardized relative secular trend estimator at age j. Additionally, denote by $\hat{\theta}_j(Y_1; Y_2)$ the estimate of θ_j obtained from participants born between years Y_1 and Y_2, and let $\widehat{SE}\left(\hat{\theta}_j(Y_1; Y_2)\right)$ refer to its estimated standard error. The estimate obtained from the bulk of participants is thus $\hat{\theta}_j(1978; 1993)$. We then calculate the following standardized differences in estimates (SDE) separately for $Y_1 \in \{1977, 1976, \dots, 1973\}$ and $Y_2 \in \{1994, 1995, \dots, 2002\}$:

$$SDE_j(Y_1) = \frac{\hat{\theta}_j(Y_1; 1993) - \hat{\theta}_j(1978; 1993)}{\widehat{SE}\left(\hat{\theta}_j(1978; 1993)\right)}$$

$$SDE_j(Y_2) = \frac{\hat{\theta}_j(1978; Y_2) - \hat{\theta}_j(1978; 1993)}{\widehat{SE}\left(\hat{\theta}_j(1978; 1993)\right)}.$$

The SDE expresses the difference between the secular trend estimate obtained from the bulk of participants and that obtained when incorporating participants born either since year Y_1 or up to year Y_2 as a function of the standard error estimated on the restricted sample. For the relative secular trend estimate, the SDE can be interpreted as a standardized difference in means (Cohen's *d*; 2), with values of 0.2, 0.5, and 0.8 referring to small, moderate, and large effects, respectively. Supplementary Figure 2 illustrates the SDE estimated when using data from participants born since year Y_1 (left column) or up to year Y_2 (right column). By definition, if $Y_1 = 1978$ or $Y_2 = 1993$, the corresponding SDE is exactly zero. We observed that including participants born before 1978 had no strong impact on the secular trend estimates with SDE remaining within ±0.5. However, including participants born after 1993 caused significant deviation in the secular trend estimates, especially in head circumference, all ages being affected, but also in full scale IQ at 9 and 14 years, with SDE sometimes reaching or exceeding one standard error. As we did not want the results to be driven by those birth years at the tail of the distribution, we restricted the main analysis to the years 1978–1993. However, this sensitivity analysis shows that reported results remain similar when including children born since 1973.

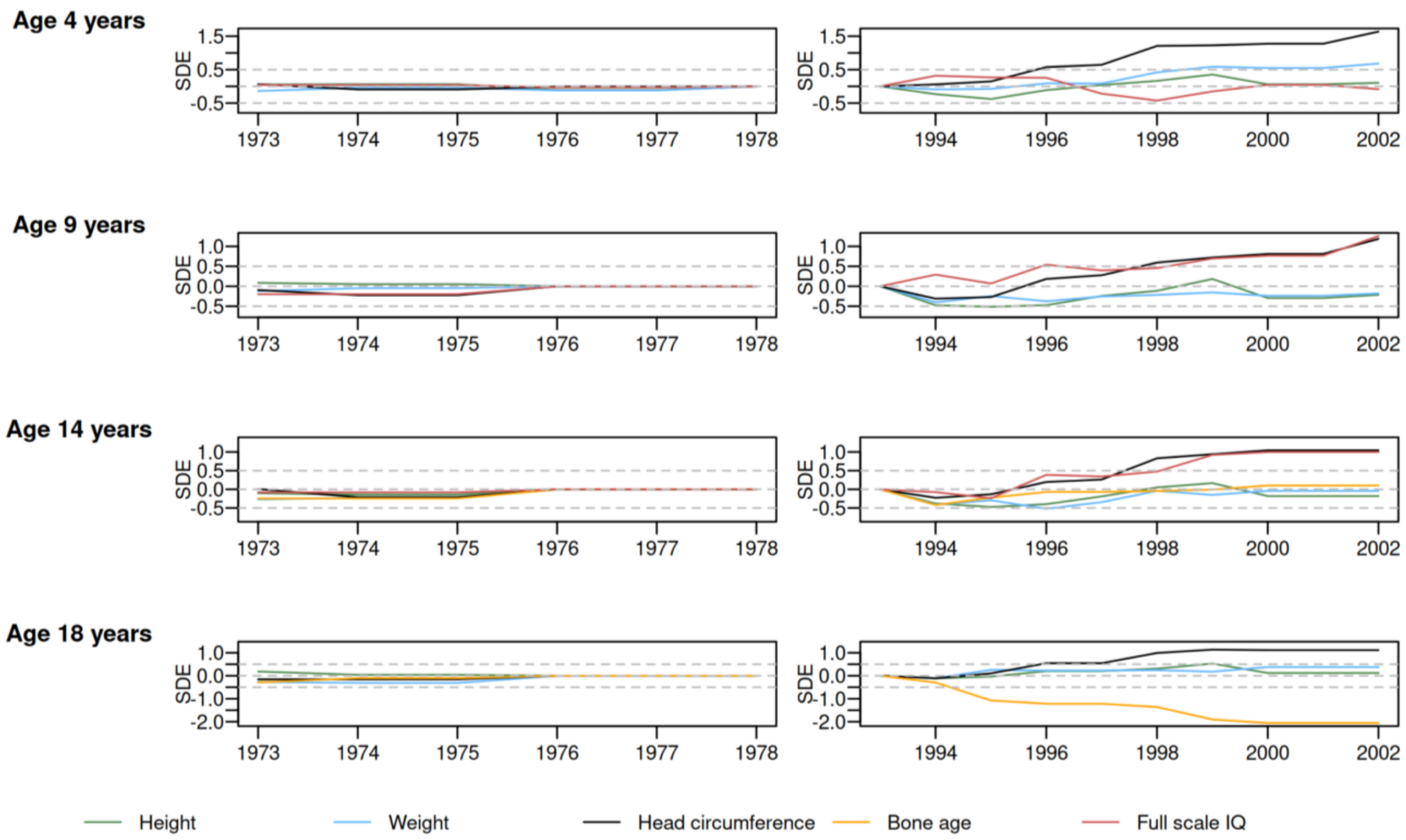

Supplementary Figure S2. Standardized difference in estimates (SDE) comparing the secular trend estimates obtained from the bulk of participants (i.e., participants born between 1978 and 1993) with those obtained by sequentially adding participants whose year of birth lies at the tails of the distribution (before 1978 or after 1993). The SDE can be interpreted as the standardized difference in means (Cohen's *d*; 2), with values of 0.2, 0.5, and 0.8 referring to small, moderate, and large effects, respectively (2). Horizontal dashed lines are placed at SDE = 0 (no difference) and SDE = ±0.5 (moderate deviation from the reference estimate).

2 Supplementary Material S2: Secular Trend Estimates Using Internal Versus External Standardization.

In this study, we used an in-sample standardization with the relative secular trend at age j defined as the ratio β_{1j}/σ_j given the model described in the methods section. With such definition, the outcome y_{ij} measured on child i at age j can refer either to an unstandardized measurement such as height in cm or to an age-standardized measure such as full-scale IQ. The term “in-sample standardization” here comes from the fact that the standard deviation σ_j used to standardize β_{1j} is estimated on the same sample: the sample under study. An alternative is to use an out-of-sample standardization procedure where the outcome value y_{ij} refers exclusively to an age-standardized value such as standard deviation score calculated using reference values from an external normative study. In this case, the secular trend at age j is simply given by β_{1j} and quantifies the magnitude of the secular trend as a function of the typical variability at age j found in the external normative sample. Both types of standardization allow the secular trend estimate to be unitless, possibly allowing interoutcome comparisons. However, each approach comes with advantages and limitations, as we now review. One advantage of the out-of-sample standardization procedure is that the definition of the secular trend is simpler and, unlike the in-sample standardization, does not require the computation of a ratio between two model parameters to obtain the estimate of interest, with additional steps required to obtain a confidence interval for such estimates. Additionally, the magnitude of the resulting secular trend estimate may be easier to interpret in some circumstances as it can be compared with a known external standard. For example, it is known that full-scale IQ in the general population has a mean of 100 and a standard deviation of 15. An externally standardized secular trend estimate of 0.5 on full scale IQ would therefore indicate that the difference in average IQ between two cohorts born 10 years apart is 0.5 × 15 = 7.5 points of IQ per decade. Considering the standard deviation as known in the external normative sample may also contribute to reducing the standard error of the secular trend estimate. Unfortunately, the advantages of externally standardized secular trend estimates come at the price of increased complexity when comparing estimates obtained from different outcomes. Although an out-of-sample standardization is perfectly suitable when all outcomes can be standardized using the same normative study, for example when comparing secular trend estimates in motor performance using components of the same motor test (3), the comparison becomes difficult when each outcome uses a different external normative sample. Indeed, in that situation, differences between relative secular trend estimates will be sensitive to intrinsic differences between the normative samples. Another problem associated with out-of-sample standardization is that the external normative sample may itself be affected by unnoticed secular trends. The presence of a secular trend in a normative sample would inflate the typical variability of the outcome reported in the normative study, which in turn would lead to an underestimation of the magnitude of the “true” secular trend in our study. An in-sample standardization “solves these two issues by using the within-cohort interindividual variability σ_j in the sample under study, with all outcomes being measured in that same sample.

Supplementary Table S1 reports relative secular trend estimates and their 95% confidence interval obtained with internal or external standardizations when using data from participants of the third cohort of the Zurich Longitudinal Studies (ZLS-3; 1) born between 1978 and 1993. External standardizations for height and weight data were performed using two normative studies. Prader et al. (4) study included participants born between 1954 and 1956, whereas the study of Eiholzer et al. (5)

study included participants born approximately between 1993 and 2019. In this way, we could investigate how the secular trend estimates in height and weight were affected by the normative sample being used in the out-of-sample standardization. For the out-of-sample standardization of intelligence measures, we used regular full-scale, verbal, and performance IQ scores as standardized outcomes, assuming a standard deviation of 15 points as in the general population. Note that performance and verbal IQ were not assessed at age 4 years. The mean of verbal IQ at 9 years is 105.0 (SD 10.9) and at 14 years, it is 108.3 (SD 12.0). The mean of performance IQ at 9 years is 101.8 (SD 11.6) and at 14 years, it is 111.2 (SD 10.0).

Although some differences can be seen, the two types of standardization provide remarkably similar results. The only notable difference is that the secular trend estimate in weight was not statistically significant both with the internal standardization and when using the Eiholzer et al. (5) study for out-of-sample standardization, but it was statistically significant when using the Prader et al. (4) study for out-of-sample standardization. However, all point estimates were close to each other, ranging from 0.25 to 0.37. Supplementary Table S1 suggests that our results are fairly robust to the type of standardization used in the calculation of relative estimates of secular trends.

Supplementary Table S1 Secular trend estimates with 95% confidence interval in square brackets at 4, 9, and 14 years for different traits and using different types of standardization for participants of the Zurich Longitudinal Studies (ZLS) born between 1978 and 1993.

Outcome	Type of standardization	Age 4 years	Age 9 years	Age 14 years	Age 18 years
Height	internal[a]	0.35 [0.00; 0.69]	0.46 [0.11; 0.82]	0.38 [0.03; 0.72]	0.09 [-0.28; 0.45]
	Eiholzer et al. (2019)[b]	0.31 [0.00; 0.63]	0.38 [0.09; 0.68]	0.36 [0.05; 0.67]	0.08 [-0.23; 0.40]
	Prader et al. (1989)[b]	0.23 [-0.08; 0.53]	0.18 [-0.08; 0.45]	0.29 [-0.01; 0.59]	-0.01 [-0.33; 0.31]
Weight	internal[a]	0.27 [-0.07; 0.61]	0.51 [0.18; 0.85]	0.51 [0.17; 0.84]	0.36 [0.00; 0.73]
	Eiholzer et al. (2019)[b]	0.25 [-0.05; 0.54]	0.46 [0.17; 0.76]	0.50 [0.19; 0.82]	0.33 [0.00; 0.65]
	Prader et al. (1989)[b]	0.37 [0.08; 0.66]	0.47 [0.19; 0.76]	0.45 [0.14; 0.75]	0.28 [-0.01; 0.57]
Head circumference	internal[a]	0.01 [-0.35; 0.37]	0.24 [-0.09; 0.58]	0.17 [-0.18; 0.53]	-0.04 [-0.41; 0.32]
	Prader et al. (1989)[b]	0.01 [-0.41: 0.42]	0.29 [-0.11; 0.70]	0.24 [-0.25; 0.73]	-0.07 [-0.61; 0.48]
Full scale IQ	internal[a]	0.54 [0.17; 0.92]	-0.54 [-0.90; -0.17]	-0.60 [-1.00; -0.21]	
	external[b]	0.39 [0.12; 0.65]	-0.35 [-0.59; -0.11]	-0.39 [-0.64; -0.14]	
Verbal IQ	internal[a]	Not applicable	-0.30 [-0.66; 0.07]	-0.85 [-1.26; -0.44]	
	external[b]		-0.18 [-0.40; 0.04]	-0.54 [-0.80; -0.28]	
Performance IQ	internal[a]		-0.61 [-0.97; -0.24]	-0.28 [-0.65; 0.09]	
	external[b]		-0.44 [-0.70; -0.18]	-0.18 [-0.42; 0.06]	

[a] The secular trend is estimated as the ratio of the difference in the outcome averages between two cohorts born 10 years apart divided by the in-sample within-cohort interindividual variability or internal standardization.
[b] The secular trend estimate quantifies the difference between two cohorts born 10 years apart in numbers of standard deviation (SD), with this SD defined on an external normative sample (external standardization). In the case of IQ, this corresponds to an SD of 15 points, as is common in IQ tests (6-8). For height and weight, we used two normative studies (4, 5). For head circumference, Prader et al. (4) study was used because this outcome was not investigated in Eiholzer et al. (5).

Supplementary Table S2 Secular trend estimates with 95% confidence interval in square brackets at 14 years unadjusted and adjusted for bone age

Outcome	Unadjusted for bone age[a]	Adjusted for bone age[b]
Height	0.38 [0.03; 0.72]	0.37 [-0.03; 0.77]
Weight	0.51 [0.17; 0.84]	0.32 [-0.07; 0.71]
Head circumference	0.17 [-0.18; 0.53]	0.22 [-0.17; 0.62]
Full scale IQ	-0.60 [-1.00; -0.21]	-0.49 [-0.94; -0.05]

[a] Secular trends quantify the contrast between two cohorts of the same age, sex, and gender born 10 years apart.
[b] Secular trends quantify the contrast between two cohorts of the same age, sex, gender and bone age born 10 years apart.

3 References

1. Wehrle FM, Caflisch J, Eichelberger DA, Haller G, Latal B, Largo RH, et al. The Importance of Childhood for Adult Health and Development-Study Protocol of the Zurich Longitudinal Studies. *Front Hum Neurosci* (2021) 14:612453. Epub 2021/02/27. doi: 10.3389/fnhum.2020.612453.

2. Cohen J. *Statistical Power Analysis for the Behavioral Sciences*. 2, editor. Hillsdale, NJ, US: Lawrence Erlbaum Associates (1988).

3. Knaier E, Chaouch A, Caflisch JA, Rousson V, Wehrle FM, Kakebeeke TH, et al. Secular Trends in Motor Performance in Swiss Children and Adolescents from 1983 to 2018. *Frontiers in Public Health* (2023) 11:971. doi: 10.3389/fpubh.2023.1095586.

4. Prader A, Largo RH, Molinari L, Issler C. Physical Growth of Swiss Children from Birth to 20 Years of Age. First Zurich Longitudinal Study of Growth and Development. *Helvetica Paediatrica Acta* (1989) 52:1-125. Epub 1989/06/01.

5. Eiholzer U, Fritz C, Katschnig C, Dinkelmann R, Stephan A. Contemporary Height, Weight and Body Mass Index References for Children Aged 0 to Adulthood in Switzerland Compared to the Prader Reference, Who and Neighbouring Countries. *Ann Hum Biol* (2019) 46(6):437-47. Epub 20191031. doi: 10.1080/03014460.2019.1677774.

6. Snijders-Oomen N. *Snijders-Oomen Nicht Verbale Intelligenztestreihe: Son 2 1/2-7*. Groningen, Netherlands: Wolters-Noordhoff (1977).

7. Tewes U, Titze I. Untersuchungen Zur Anwendung Des Hawik in Der Klinischen Und Sonderpädagogischen Diagnostik [Studies on the Application of Hawik in Clinical and Special Educational Diagnosis]. *Zeitschrift für Differentielle und Diagnostische Psychologie* (1983) 4:179-201.

8. Willich O, Friese H. Der Hamburg-Wechsler-Intelligenztest Für Kinder Revision 1983 (Hawik-R). *Diagnostica* (1994) 40:172-89.
